# The effects of electroconvulsive therapy on cognition: an exploratory retrospective study

**DOI:** 10.1017/S1092852925100606

**Published:** 2025-10-10

**Authors:** Susanna Riessland, Pia Baldinger-Melich, Nicoletta Margreiter-Neuwirth, Ursula Kainzmayer, Ina Bozic, Rupert Lanzenberger, Richard Frey, Dan Rujescu, Vincent Millischer

**Affiliations:** 1 Department of Psychiatry and Psychotherapy, Clinical Division of General Psychiatry, Medical University of Vienna, Spitalgasse 23, 1090 Vienna, Austria; 2 Comprehensive Center for Clinical Neurosciences and Mental Health, Spitalgasse 23, 1090 Vienna, Austria; 3 Department of Molecular Medicine and Surgery, Karolinska Institutet, 171 77 Stockholm, Sweden; 4 Center for Molecular Medicine, Karolinska University Hospital, Visionsgatan 18, 171 76 Stockholm, Sweden

**Keywords:** Electroconvulsive therapy, cognition, depression, treatment, cognitive side effects, treatment-resistant depression

## Abstract

**Objectives:**

Electroconvulsive therapy (ECT) is one of the most effective treatments for depression, but worries about cognitive side effects remain. This retrospective study evaluated cognitive outcomes and the antidepressant efficacy of ECT in a real-life sample of patients with treatment-resistant uni- or bipolar depression.

**Methods:**

We included 90 depressed inpatients aged 49 ± 13.8 (SD) years who underwent 10 ± 2.1 (SD) unilateral or bitemporal ECT treatments and completed an extensive pre- and post-treatment psychological test battery. The Hamilton Depression Rating Scale (HAMD) and the Mini-Mental State Examination (MMSE) were evaluated as main outcomes pre-/post-ECT treatment.

**Results:**

There was no significant change in MMSE scores between pre-/post-treatment assessments (*β* = 0.10, 95% confidence interval [CI] [−0.44, 0.25], *p* = 0.58), indicating no negative effect on global cognition. A minority of patients (*N* = 3) experienced a reduction of ≥5 points in the MMSE. Most cognitive tests showed no difference; however, some domains revealed statistically significant improvements (visual learning and motoric reaction time), whereas one domain showed a significant decline (verbal learning). Higher age and higher stimulus doses predicted worse outcomes in some cognitive domains. While ECT significantly reduced depressive symptoms measured by HAMD (*β* = −5.51, 95% CI [−7.08, −3.94], *p* < 0.001), depressive symptoms were not associated with cognitive outcomes.

**Conclusions:**

No major cognitive changes were observed. While test results indicated deterioration in verbal learning and improvement in visual learning and motoric reaction time, effect sizes were small, and other cognitive tests showed no significant changes. The main limitation is the absence of retrograde memory assessment.

## Background

Electroconvulsive therapy (ECT) is an indispensable treatment option for severe, life-threatening cases of psychiatric illness. It is a highly effective treatment for depression,^1^ including treatment-resistant depression (TRD),^2^ and may outperform traditional pharmacological antidepressant therapies.^3^ ^-^^5^ Nevertheless, ECT remains underutilized partly due to misinformation and persistent stigma.^6^ This includes the fear of cognitive impairments, which are regarded as one of the most concerning side effects of ECT. In a recent meta-analysis focusing on subjective aspects of cognition following ECT, almost half of the patients reported cognitive side effects.^7^ However, the objective assessment of these impairments has proven to be more complex.

Cognitive impairments by ECT can be divided into different categories: non-memory cognition, anterograde memory, and retrograde memory.^8^ Non-memory cognition includes global cognitive function (eg, measured by the Mini-Mental State Examination [MMSE]),^9^ attention (eg, measured by digit span forward test),^10^ processing speed (eg, assessed by the Trail Making Test—Part A [TMT Part A]), and executive functioning (eg, assessed by the TMT Part B).^11^ Anterograde memory can be measured by impairment of new learning by a verbal learning test with delayed recall.^12^ Retrograde memory includes autobiographical memory, which is most often assessed by the Columbia University Autobiographical Memory Interview—Short form (CUAMI-SF) or CUAMI.^13^ To characterize cognitive complaints, a range of tests is, therefore, necessary to assess the various aspects of cognition and help to objectify complaints of cognitive deficits by patients.^8^

Since depression, ECT, and anesthesia can all cause impairment in different cognitive domains, it is challenging to disentangle the origin of cognitive deficits.^14^ ^-^^16^ In depression, processing speed, executive/working memory, verbal learning, and memory are the domains most impaired, with some patients also experiencing global cognitive impairment.^17^ In recurrent depressive disorder—one of the main indications for ECT—cognitive deficits are more pronounced than in a first depressive episode. When depressive symptoms resolve, some cognitive deficits commonly improve (eg, psychomotor speed), whereas other neuropsychological domains (eg, verbal/nonverbal learning and memory, executive function) often remain impaired.^14^ ^,^^17^ Certain cognitive deficits following ECT might therefore be attributable to depression, even when symptoms have improved, and particularly when depressive symptoms persist.

Regarding ECT, a recent meta-analysis showed no deterioration in global cognitive function measured by MMSE directly post-ECT,^18^ whereas another meta-analysis found slight impairment in the first 3 days post-ECT, but improvement to baseline thereafter.^15^ In addition, in late-life depression, patients undergoing ECT showed no decline directly or 6 months post-ECT in MMSE scores, with patients who performed worse at baseline even showing an overall improvement.^19^ ^,^^20^ However, important inter-individual variability exists, with a minority of elderly patients experiencing a deterioration of their global cognition post-ECT.^19^

Focusing on specific domains, certain areas of non-memory cognition, including processing speed and executive functioning, are impaired subacutely up to 3 days post treatment, while attention and working memory, visual episodic memory, spatial problem solving, and intellectual ability remain comparable to baseline. More than 3 days after ECT completion, most non-memory cognitive functions showed at least slight improvement beyond baseline, whereas none showed a deterioration.^15^ Verbal fluency and verbal episodic memory showed a decline until 2 to 4 weeks after ECT, but then returned to baseline levels, indicating no long-term effect on anterograde memory.^15^ ^,^^18^ Several studies found impairment in retrograde autobiographical memory, with persistence of impairment in a subset of patients at a 6-month follow-up after ECT, using CUAMI-SF or CUAMI.^21^ ^-^^23^ While autobiographical memory impairment post-ECT objectively improved over time, with nearly complete resolution between 1 month^18^ and 6 months, patients report persistent amnesia beyond this period.^24^

Multiple factors have been associated with cognitive side effects post-ECT: patient-specific characteristics, treatment parameters like electrode placement, stimulus charge, and number of treatments.^8^ For electrode placement, bitemporal (BT) and right unilateral (RUL) are most often used—the former being commonly associated with better, faster response but more pronounced cognitive impairments, whereas the latter is considered less effective, but results in fewer cognitive deficits.^1^ In order to achieve an optimal antidepressant response while concomitantly mitigating the risk of cognitive deficits, it is imperative to assess both treatment parameters and patient characteristics. However, the significance of different factors remains not fully understood.

Given the heterogeneity of existing studies regarding cognition, we aimed to retrospectively examine cognitive side effects in a real-world cohort of depressed patients undergoing ECT. Furthermore, we explored the influence of demographics, treatment parameters, and seizure parameters on the effects of ECT on depressive symptoms and cognition.

## Materials and methods

### Subjects

Data of all patients aged 18 and older undergoing an ECT series (>6 sessions) between January 1, 2016, and February 14, 2024, at the Department of Psychiatry and Psychotherapy, Medical University of Vienna, were screened. Patients treated for depressive symptoms diagnosed with unipolar or bipolar depression (International Classification of Diseases [ICD]-10, F31.3, F31.4, F31.5, F32.1, F32.2, F32.3, F33.1, F33.2, and F33.3) who also completed routine psychological testing before and after the ECT series were included in the study. Data from maintenance or continuation ECT treatments were excluded from the analysis. This retrospective study was approved by the Ethics Committee of the Medical University of Vienna (EK No. 1161/2024) and performed in accordance with the Code of Ethics of the World Medical Association (Declaration of Helsinki).

### ECT treatment

All ECT sessions were performed with the Thymatron System IV (Somatics LLC, Lake Bluff, Illinois) ECT device using brief-pulse, square-wave, constant current. Patients underwent treatment with RUL or BT electrode placement. ECT was administered until remission was achieved or until no further improvement was anticipated. The titration method was utilized to reach an electrical stimulus dose that resulted in an adequate seizure.^25^ For patients who did not show sufficient response after 5 to 6 RUL treatments, treatment modality was switched from RUL to BT electrode placement. Likewise, the modality was switched from BT to RUL stimulation in patients who showed clinically substantial cognitive side effects. For capacity reasons and to ensure faster response, the standard stimulation technique in our department was switched on January 1, 2020, from thrice-weekly RUL to twice-weekly BT stimulation.

### Variables and measures

A neuropsychological test battery was routinely performed in German by experienced clinical psychologists before and after the ECT series in depressed patients that included a wide range of cognitive tests: MMSE,^9^ Non-Verbal Learning Test (NVLT), Verbal Learning Test (VLT),^26^ Cognitrone S1,^27^ Vienna Reaction Test (RT) S5,^28^ and TMT Part A and Part B S1.^11^ The cognitive domains measured by these assessments are summarized in Table 1. Furthermore, the following depression-related outcomes were included in the test battery: the Beck Depression Inventory (BDI-II),^29^ Hamilton Depression Rating Scale—17 items (HAMD),^30^ Symptom Checklist 90-R—Depression (SCL-R) scale,^31^ and, from April 22, 2021 onward, Montgomery–Åsberg Depression Rating Scale (MADRS).^32^ Additionally, state and trait anxiety were measured by the State–Trait Anxiety Inventory (STAI).^33^

**Table 1.** Cognitive Domains of the Neuropsychological Test Battery

Domain	Neurocognitive measures	Metric	Effect on cognition (↑ = better, ↓ = worse)
Global cognitive functioning screening	MMSE	Number of correct responses	More points = ↑
Non-verbal learning	NVLT	∑(Correct − Incorrect Responses)	More points = ↑
Verbal learning	VLT	∑(Correct − Incorrect Responses)	More points = ↑
Attention and concentration	Cognitrone	Seconds to correct rejection	Longer time = ↓
Motoric reaction time	RT-Motoric	Seconds	Longer time = ↓
Decision reaction time	RT-Decision	Seconds	Longer time = ↓
Processing speed	TMT-A	Seconds	Longer time = ↓
Executive functioning	TMT-B	Seconds	Longer time = ↓

Data for each treatment relative to patient information (patient ID, age, gender, and diagnosis) and ECT treatment (RUL/BT, seizure adequacy, charge delivered, EEG, and EMG endpoints) were obtained from the electronic health records of our clinic.

### Statistical analyses

**Figure 1.**
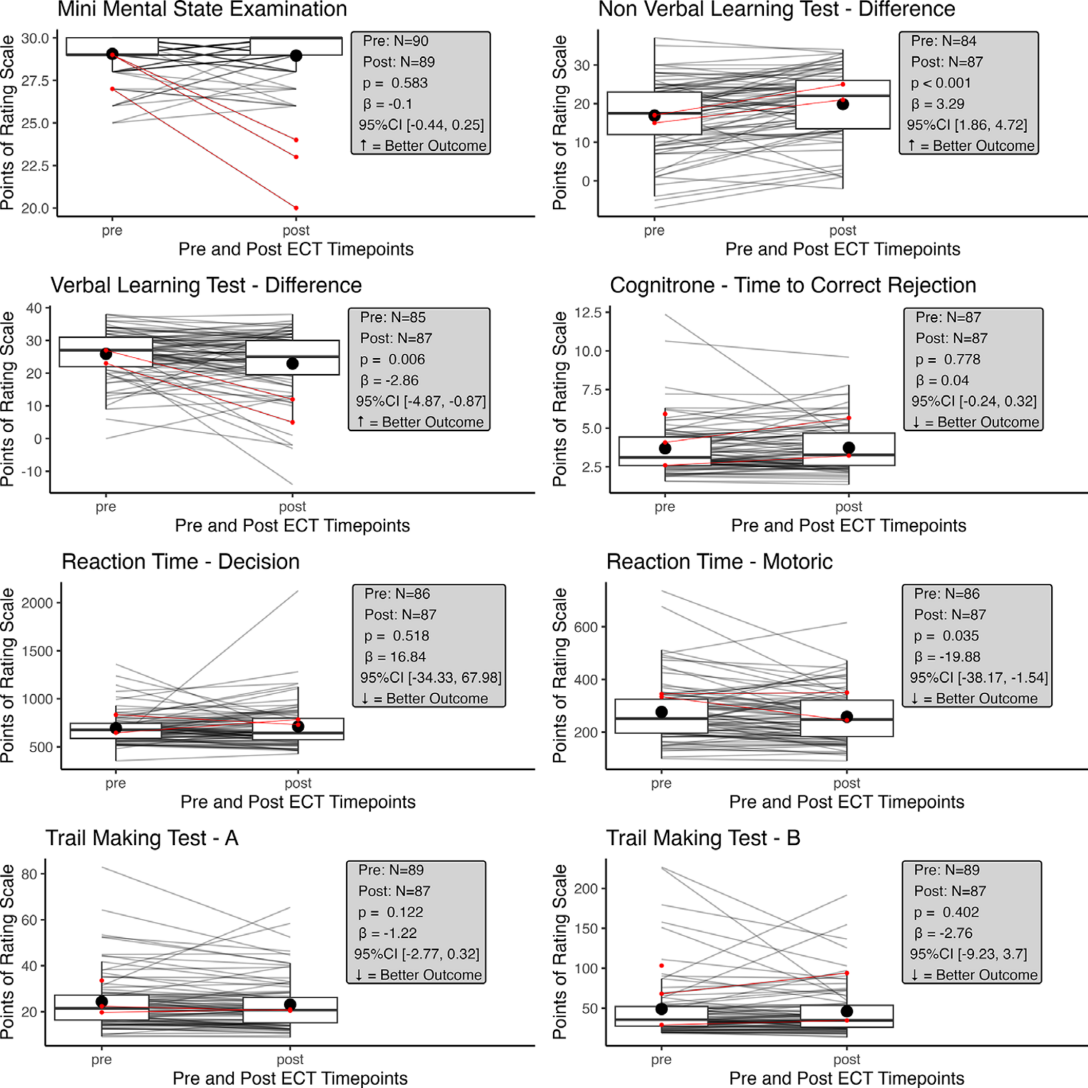
Results from cognitive tests pre-/post-ECT. The central line within each box indicates the median value. The box limits illustrate the interquartile range (IQR; from the 25th to the 75th percentile). The whiskers extend to the minimum and maximum values within 1.5 times the IQR from the quartiles. Data points beyond whiskers are shown as lines. The large black dot indicates mean scores. Each gray line represents individual patients’ scores over time, with those outlined in red showing the 3 patients with deterioration in MMSE scores post-ECT. The number of observations pre-/post-ECT, *p*-value, *β*-estimate, 95% CI, and whether an increase/decrease signifies a better outcome are shown in the gray box for each test.

All statistical analyses were performed using R (Version R-4.3.2). Linear mixed-effects models were performed to compare cognitive and depressive variables pre-/post-ECT using the lmer() function from the lme4 package,^34^ in order to better account for missing data (see Figure 1 and Supplementary Figure 1). Cognitive and depressive test results were entered as dependent variables, time point was entered as fixed effect, and patient ID as a random effect. Coefficients (beta-estimates) and *p*-values were extracted using the summary() function, and 95% confidence intervals (CIs) were calculated using the confint() function, all from the stats package. To assess the representativeness of our analyzed group (ie, patients with pre-/post-testing available) among all ECT patients at our center, we used independent-samples *t*-tests with equal variances to compare (1) Clinical Global Impression (CGI) severity scores between patients with and without psychological testing, and (2) pre-treatment variables between patients with only pre-ECT assessments and those with both pre- and post-ECT assessments. These comparisons aimed to identify any differences in disease severity or cognitive impairment between groups.

In our exploratory analysis, we aimed to evaluate the relationships between cognitive outcomes and explanatory factors previously reported to influence ECT tolerability. These factors were age, gender, pre- and post-ECT HAMD scores, days from the last ECT session to cognitive testing, the proportion of BT treatments in a series, psychotic symptoms, mean number of ECT treatments per week, uni- versus bipolar depression, the number of ECT treatments, the mean stimulus dose (charge), and EEG and EMG mean durations. A linear regression model was fitted for each combination of outcome and explanatory variables using the lm() function from the stats package. Post-ECT test results were used as dependent variables, whereas the aforementioned explanatory factors and pre-ECT test results of the same outcome were included as predictors. To account for potential confounding and the interrelationships between predictors, we subsequently fitted multivariable linear regression models incorporating all explanatory variables simultaneously. Beta-estimates, 95% CI, and *p*-values for each predictor were calculated as stated above. Finally, we analyzed the effects of different stimulation combinations (RUL, RUL-to-BT, and BT) using a linear regression model as described above, followed by pairwise group comparisons using the emmeans package^35^ with Tukey adjustment for multiple testing. BT-to-RUL was excluded from the analysis due to the small sample size (*n* = 3). The alpha level of significance was set at 0.05. Due to the exploratory nature of the study, no adjustment for multiple testing was performed.

## Results

### Demographics and clinical characteristics

A total of 90 patients were included in the analysis. The demographic characteristics of the study population are summarized in Table 2. The mean age of the participants was 49 (SD 13.8) years; 42% identified as men and 58% as women. On average, participants underwent 10 (SD 2.08; range: 6-16) ECT sessions. The vast majority of patients had unipolar depression (90%), most had severe depression without psychotic symptoms (83%), and were treated with bilateral electrode placement (54%). An average of 5 days (SD 4.16; range: 1-21) passed between the last ECT and cognitive testing. In 5.3% of ECT sessions, restimulation was performed due to insufficient seizure quality.

**Table 2.** Demographics and Clinical Characteristics

	BT (*N* = 49)	BT-to-RUL (*N* = 3)	RUL-to-BT (*N* = 25)	RUL (*N* = 13)	All (*N* = 90)
Age					
Mean (SD)	45.4 (14.3)	49.3 (8.08)	51.5 (13.6)	55.4 (9.92)	48.7 (13.8)
Min, max	21.0, 80.0	42.0, 58.0	22.0, 79.0	35.0, 72.0	21.0, 80.0
Sex					
Male	20 (40.8%)	2 (66.7%)	10 (40.0%)	6 (46.2%)	38 (42.2%)
Female	29 (59.2%)	1 (33.3%)	15 (60.0%)	7 (53.8%)	52 (57.8%)
Number of ECT in a series					
Mean (SD)	9.61 (1.58)	9.67 (1.53)	11.4 (2.16)	8.62 (2.33)	9.98 (2.08)
Min, Max	6.00, 14.0	8.00, 11.0	7.00, 16.0	6.00, 14.0	6.00, 16.0
Severity of depression					
Moderate	6 (12.2%)	0 (0%)	1 (4.0%)	0 (0%)	7 (7.8%)
Severe without psychotic symptoms	42 (85.7%)	3 (100%)	20 (80.0%)	10 (76.9%)	75 (83.3%)
Severe with psychotic symptoms	1 (2.0%)	0 (0%)	4 (16.0%)	3 (23.1%)	8 (8.9%)
Type of diagnosis					
Unipolar	44 (89.8%)	3 (100%)	22 (88.0%)	12 (92.3%)	81 (90.0%)
Bipolar	5 (10.2%)	0 (0%)	3 (12.0%)	1 (7.7%)	9 (10.0%)
Days from last ECT to cognitive testing					
Mean (SD)	4.41 (4.27)	1.33 (0.58)	6.92 (4.15)	4.46 (2.79)	5.01 (4.16)
Min, Max	1.00, 18.0	1.00, 2.00	2.00, 21.0	1.00, 10.0	1.00, 21.0
Response HAMD–17					**(*N* = 87)**
Yes	2 (66.7%)	12 (24.5%)	6 (24.0%)	8 (61.5%)	28 (31.1%)
No	1 (33.3%)	37 (75.5%)	17 (68.0%)	4 (30.8%)	59 (65.6%)
Remission HAMD–17					**(*N* = 87)**
Yes	1 (33.3%)	10 (20.4%)	5 (20.0%)	8 (61.5%)	24 (26.7%)
No	2 (66.7%)	39 (79.6%)	18 (72.0%)	4 (30.8%)	63 (70.0%)

*Note:* BT-to-RUL indicates switch to RUL because of cognitive side effects, and RUL-to-BT shows switch after 5 to 6 RUL ECTs due to the lack of partial response.

### Population representativeness

A total of 226 depressed patients completed an ECT series during the study period, of whom 133 (59%) underwent psychological testing. In the subsample with available pre-ECT CGI severity scores (*n* = 144), patients without testing had higher baseline severity, with a mean CGI of 5.68 versus 5.25 (*t*(142) = 2.52, 95% CI [0.09, 0.77], Cohen’s *d* = 0.42, *p* = 0.013), which may have limited participation in psychological assessment.

Of the 133 patients with psychological testing, 37 (28%) completed only the pre-ECT assessment and were excluded from pre-/post-analyses. Those patients had significantly higher HAMD-17 scores (*t*(121) = −2.79, 95% CI [−5.77, −0.98], Cohen’s *d* = −0.61, *p* = 0.006) and worse performance in MMSE (*t*(127) = 2.60, 95% CI [0.21, 1.54], Cohen’s *d* = 0.53, *p* = 0.010), VLT (*t*(115) = 2.15, 95% CI [0.28, 6.81], Cohen’s *d* = 0.48, *p* = 0.034), TMT-A (*t*(122) = −2.31, 95% CI [−13.13, −1.02], Cohen’s *d* = −0.50, *p* = 0.022), and Cognitrone (*t*(120) = −2.03, 95% CI [−1.68, −0.02], Cohen’s *d* = −0.44, *p* = 0.044), indicating worse cognitive performance pre-ECT. All other cognitive and depressive variables did not show any significant differences pre-ECT. This likely reflects that more severely ill patients are more likely to drop out of post-testing with minor cognitive worsening, although discharge before follow-up (patient-initiated or administrative) likely also played a role.

### Treatment response

Patients showed a significant treatment response through reductions in depression scores measured by HAMD (*β* = −5.51, 95% CI [−7.08, −3.94], *p* < 0.001), which was also observed in other scales for depression, including self-rating scales (Supplementary Figure 1 and Supplementary Table 1). Anxiety levels, measured with the STAI, also declined in both trait (*β* = −9.14, 95% CI [−11.78, −6.51], *p* < 0.001) and state (*β* = −10.22, 95% CI [−12.91, −7.52], *p* < 0.001) between both assessments.

### Cognition

No statistically significant difference was observed in global cognitive ability between the 2 time points as measured by the MMSE (*β* = 0.10, 95% CI [−0.44, 0.25], *p* = 0.58). Similarly, the majority of the more specific cognitive variables tested did not change. However, there was a statistically significant difference in the NVLT as false positives decreased (*β* = −3.39, 95% CI [−4.96, −1.80], *p* < 0.001), and therefore the difference between correct and false sum scores increased after ECT (*β* = 3.29, 95% CI [1.86, 4.72], *p* < 0.001). This indicates an improvement in visual learning following treatment. VLT scores, on the other hand, deteriorated slightly across all domains; the difference between correct and false sum scores (*β* = −2.86, 95% CI [−4.87, −0.87], *p* = 0.006) shows a decrease in verbal learning post-ECT. Motoric reaction time improved slightly (*β* = −19.88, 95% CI [−38.17, −1.54], *p* = 0.035), whereas decision time until reaction did not significantly change after treatment. The Cognitrone test, which measures attention and concentration, showed no difference between time points. Likewise, no statistical difference was observed in processing speed measured by the TMT Part A and executive functioning measured by the TMT Part B. The results are shown in Figure 1 (for details, see Supplementary Table 2).

### Description of individuals with important deterioration

Even though, at the group level, no major changes in global cognition were observed, 3 patients showed an important deterioration of MMSE scores (≥5 points) in the pre-post comparison. One patient had psychological testing performed only hours after ECT and, therefore, might have had more pronounced deficits (29 to 23 points). Another patient whose score deteriorated substantially (29 to 24 points) had testing 1 day after the last ECT at the day of their discharge. A third patient with a strong deterioration (27 to 20 points) was tested 3 days after their last ECT and was unable to perform tests other than the MMSE post-ECT due to a lack of concentration. The outcomes of these 3 patients in the other cognitive tests are visualized by red dots in Figure 1. None of these patients was delirious during testing, and selective attention was available.

### Exploratory part

**Figure 2.**
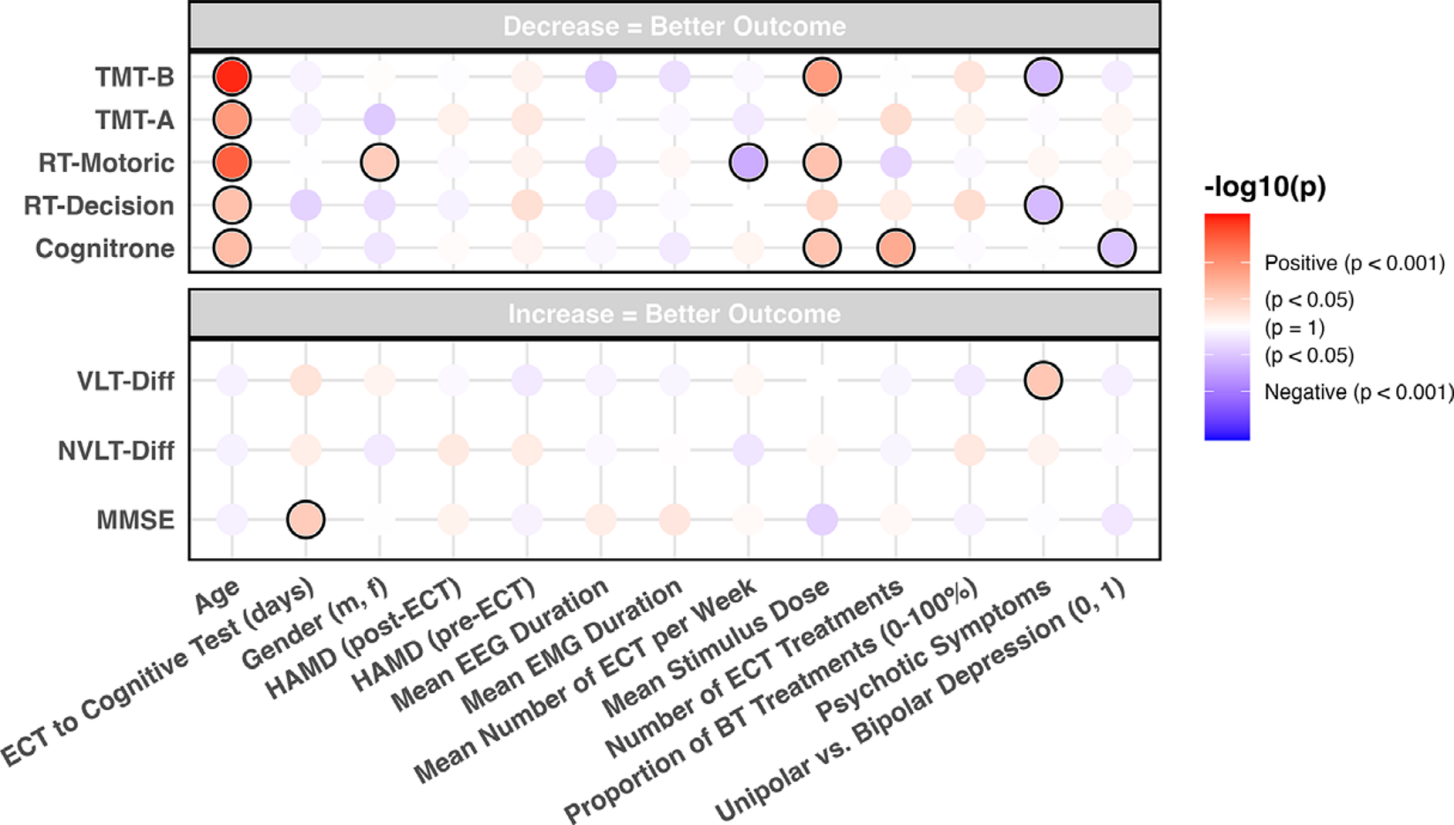
Associations between post-ECT cognitive and predictive variables. Each variable is corrected for pre-ECT values. Red indicates a positive association, blue indicates a negative association, and the darkness indicates the strength of the association. In the 5 variables shown above, a decrease (in seconds) equals a better outcome, whereas in the 3 variables shown below, an increase (in points) indicates a better outcome. Significant results (*p*-value < 0.05) are indicated by a black circle.

In an exploratory approach, we analyzed the effects of variables commonly associated with treatment response or cognitive side effects on post-ECT cognitive outcomes after adjustment for pre-ECT test results. (Figure 2 and Supplementary Table 3).

MMSE scores increased for each additional day between the last ECT session and cognitive testing (*β* = 0.08, 95% CI [0.00, 0.16], *p* = 0.040), indicating a positive effect of time elapsed since ECT on global cognitive functioning. However, when performing a sensitivity analysis by removing the 3 patients with a significant decline (see above), this association was no longer significant (*β* = 0.04, 95% CI [−0.01, 0.91], *p* = 0.158). Higher mean stimulus dose was significantly associated with higher time to correct rejection in Cognitrone (*β* = 0.01, 95% CI [0.00, 0.02], *p* = 0.023), slower motoric reaction time (*β* = 0.56, 95% CI [0.09, 1.04], *p* = 0.021), and longer completion times for TMT-B (*β* = 0.24, 95% CI [0.09, 0.40], *p* = 0.002) post-ECT, adjusted for pre-ECT test results. The number of ECT treatments was significantly associated with longer time to correct rejection in the Cognitrone (*β* = 0.17, 95% CI [0.05, 0.29], *p* = 0.005).

Psychotic symptoms predicted better outcomes on multiple measures: VLT (*β* = 9.08, 95% CI [0.93, 17.22], *p* = 0.029), faster decision times on RT-Decision (*β* = −236.46, 95% CI [−446.05, −26.87], *p* = 0.028), and shorter completion times on the TMT-B (*β* = −23.28, 95% CI [−43.89, −2.66], *p* = 0.027). Bipolar depression was associated with superior performance on the Cognitrone task (*β* = −0.88, 95% CI [−1.74, 0.01], *p* = 0.047). A higher mean number of ECT sessions per week was associated with faster motoric reaction times on RT-Motoric (*β* = −60.23, 95% CI [−107.69, −12.77], *p* = 0.014).

Older age was associated with worse cognitive performance post ECT across multiple domains: with a longer time to correct rejection in the Cognitrone (*β* = 0.02, 95% CI [0.00, 0.04], *p* = 0.015), a longer decision time until reaction (*β* = 4.55, 95% CI [0.74, 8.36], *p* = 0.020), and a slower motoric reaction time (*β* = 2.51, 95% CI [1.30, 3.71], *p* < 0.001). Age was also associated with longer completion times for both TMT-A (*β* = 0.18, 95% CI [0.07, 0.29], *p* = 0.002) and TMT-B (*β* = 0.86, 95% CI [0.50, 1.23], *p* = 0.000). Furthermore, female gender was associated with slower motoric reaction time (*β* = 32.66, 95% CI [1.41, 63.90], *p* = 0.041). Of note, the proportion of BT treatments within a series, the mean EEG and EMG durations, and pre- or post-depression severity (measured by HAMD) were not significantly associated with any of the cognitive variables post-ECT. When all explanatory variables were incorporated simultaneously in the model as explanatory variables, most results remained similar. However, the associations between MMSE and ECT to Cognitive Test (days), as well as VLT, RT-Decision, TMT-B, and psychotic symptoms, were no longer significant, while better performance on the TMT-B for patients with bipolar depression became significant (for details, see Supplementary Figure 2 and Supplementary Table 4).

Comparing different electrode placements, the only significant difference we found was the RUL group to have a faster RT-Decision compared to the RUL-to-BT (*β* = −155 milliseconds, SE = 52.3, *p* = 0.011). No other significant differences emerged among RUL, RUL-to-BT, and BT groups. We excluded BT-to-RUL due to limited sample size, so no conclusions can be drawn regarding this group.

### Sensitivity analysis

To ensure that our results were not influenced by the 3 outliers discussed above, we performed a sensitivity analysis for all analyses on the remaining sample. After the removal of these outliers, only the association between time after cognitive testing and MMSE score was no longer significant, as discussed above. For all other main and exploratory tests, the significance was not influenced.

## Discussion

In this retrospective study, we found no major effect of ECT treatment on cognition assessed by a broad neuropsychological test battery. While the results revealed a heterogeneous pattern, most tests showed no significant changes between pre- and post-ECT assessments. Most importantly, no statistically significant change in global cognition, measured by MMSE, between the pre- and the post-assessment was observed. This is in line with recent findings by Obbels et al.^20^ in late-life depression and the meta-analyses of Semkovska and McLoughlin and Landry et al.^15^ ^,^^18^ who reported no impairment of MMSE after 3 days post-ECT. The 3 patients in our sample who experienced a decline post treatment were tested within 3 days post-ECT, where a slight deterioration in MMSE scores has been reported by Semkovska and McLoughlin.^15^ Global cognition is primarily acutely impaired, where short-term anesthesia-related cognitive impairments could also have an impact.^16^

Most specific cognitive domains were also not affected by ECT treatment. While attention and concentration, processing speed, and executive functioning remained unchanged over time, in line with the literature,^36^ NVLT scores indicate a significant improvement in visual learning after ECT. This stands in contrast to the findings of Landry et al., who reported no significant changes, and Semkovska and McLoughlin, who described small acute short-term impairments in non-verbal learning. Yet, both studies detected small long-term improvements.^15^ ^,^^18^ Furthermore, we showed that verbal learning as measured by VLT deteriorated significantly, replicating the results of 2 previous studies.^37^ ^,^^38^ However, another study found no impairment in verbal learning and memory post-ECT,^39^ and improvements reported in long-term follow-up studies^15^ ^,^^18^ indicate only short-term impairment in verbal learning. Finally, motoric reaction time also improved post-ECT. While psychomotor speed impairment can be caused by depression and improves with amelioration of depressive symptoms,^14^ we did, however, not see an association between post-ECT motoric reaction time and depressive symptoms.

In our exploratory analysis, we found higher mean stimulus doses associated with worse outcome in some tests (Cognitrone, RT-Motoric, and TMT-B), whereas mean EEG and EMG duration showed no association with any cognitive variables. Older age was significantly associated with worse performance post-ECT in a number of cognitive tests (RT-Motoric, RT-Decision, Cognitrone, TMT-A, and TMT-B), whereas a meta-analysis found better cognitive outcomes with increasing age.^7^ Better performance of psychotic patients in some tests (VLT, RT-Decision, and TMT-B) could be explained by greater amelioration following ECT, consistent with evidence that psychotic symptoms predict good treatment response.^40^ Bipolar patients performed better in the Cognitrone. A higher mean weekly ECT frequency was linked to faster RT-Motoric, although increased treatment frequency has been associated with greater cognitive side effects.^41^ Finally, our analysis did not reveal an association between the proportion of BT treatments and worse cognitive outcomes after ECT, which contrasts with the literature, where BT stimulation generally has a faster, higher response with increased risk of cognitive side effects compared to RUL.^1^ However, another study observed similar remission rates for all electrode placements with similar cognitive side effects,^42^ and a recent meta-analysis found that high-dose RUL had similar response rates compared to BT ECT but fewer cognitive side effects.^43^ In addition, more treatments and higher electric stimulus have been reported to result in more cognitive impairments.^44^ When accounting for covarying factors in the explanatory model of cognitive variables, slight differences were found. Associations between MMSE and ECT to Cognitive Test (days), as well as VLT, RT-Decision, TMT-B, and psychotic symptoms, were no longer significant, while better performance on the TMT-B for patients with bipolar depression became significant. These changes are not surprising, given the liberal alpha threshold used in the exploratory analyses.

In our comparison of electrode placement combinations (RUL, RUL-to-BT, and BT), patients receiving RUL stimulation showed faster RT-Decision times than the RUL-to-BT group, possibly due to greater illness severity or a higher number of treatments needed in the latter. No other significant differences were found between combinations. The exploratory findings should, however, be interpreted with caution, due to the limited sample size and multiple testing.

All depression scores (HAMD, MADRS, BDI-II, and SCL-90 Depression) demonstrated a clear reduction, corroborating the extensive literature supporting the efficacy of ECT in treating depression.^1^ Likewise, anxiety in state and trait (STAI) declined significantly, confirming recent literature that shows a decrease in anxiety, even though it is slower and less pronounced than for depressive symptoms.^45^ ^,^^46^ The observed reduction in depressive symptoms was, however, less pronounced than previously reported. This discrepancy may be attributable to our tertiary care facility treating severely ill, treatment-refractory patients, many of whom present with comorbid psychiatric disorders, including trauma-related or personality disorders. These factors have been shown to decrease the efficacy of ECT.^47^ ^,^^48^

### Limitations

A key limitation of this retrospective data analysis is the absence of specific tests assessing retrograde, more specifically autobiographical, memory, as they were not implemented in the routinely performed psychological pre-/post-ECT assessments at our department. This precludes any conclusions regarding potential cognitive impairments in this domain, with research having found autobiographical memory decline after ECT.^18^ ^,^^39^ We did not examine verbal fluency, another specific cognitive domain that recent studies found to decline^36^ ^,^^37^; one study also associated with higher electrical stimulus in RUL.^36^ Another limitation is that there was no long-term follow-up to assess cognitive changes over time, such as after 3, 6, or 12 months, even though, as seen in the literature, many cognitive deficits are present only shortly after ECT, with improvements in almost all cognitive domains seen after 1 month.^15^ ^,^^18^ ^,^^37^ As noted in our results on population representativeness, patients with more severe symptoms were less likely to complete both pre- and post-treatment psychological assessments. This potential bias should be carefully considered when interpreting our findings, as it may lead to a more conservative estimate of treatment effects across the full spectrum of disease severity.

Our main cognitive outcome measure, the MMSE, was not developed to measure cognition following ECT but rather as a screening tool for dementia, measuring global cognitive function. Although the MMSE is used frequently in ECT literature, this can be viewed as a limitation, as it has low sensitivity for the decline of cognition in verbal recall or verbal fluency post-ECT.^49^ Therefore, instead of MMSE or the Montreal Cognitive Assessment (MoCA), other assessments such as the ElectroConvulsive therapy Cognitive Assessment (ECCA), the Autobiographical Memory Test (AMT), or the Screen for Cognitive Impairment in Psychiatry (SCIP) could be used in the future, as they might be more sensitive to detect more subtle cognitive deficits following ECT treatment. The ECCA can be administered in under 10 minutes and includes questions to test autobiographical memory, attention, temporal orientation, verbal delayed recall, and factual knowledge. Hermida et al.^50^ found a significant deterioration in ECCA scores compared to MoCA before the sixth and last ECT treatment, while pre-ECT scores had been similar. Recently, the Chinese version of the ECCA was validated, where significant differences between ECCA pre- and post-ECT treatment scores were observed, whereas there was no significant change in pre- versus post-MoCA scores.^51^ Both the AMT—which measures primarily autobiographical memory—and the SCIP—which measures immediate and delayed verbal learning, working memory, verbal fluency, and processing speed—have been shown to be sensitive to detect cognitive deficits in ECT patients.^52^ ^,^^53^

In the present study, results across the 2 exploratory modeling approaches were largely similar, suggesting a degree of robustness of the observed associations. However, given the liberal alpha threshold applied in these analyses, the findings should be interpreted with caution. We present these exploratory results to provide a foundation for future research rather than serving as confirmatory evidence. Another limitation, this being a retrospective data analysis, is the potential bias related to the use of concomitant antidepressant medication.

### Conclusion

Due to persistent fears of cognitive impairment and stigma, depressed patients struggle to access optimal care, including ECT. However, in our sample, apart from verbal learning impairment, and improvement of non-verbal learning and motoric reaction time, we did not find significant cognitive changes after an acute RUL (performed thrice weekly) or BT (performed twice weekly) ECT series. Though a minority of patients may experience a cognitive decline shortly after ECT, on the group level, we found no major cognitive side effects, indicating that it is overall a safe treatment in severely depressed patients. Future research should conduct cognitive assessments at a standardized time point (eg, 7 days) following the last ECT to increase comparability across studies and minimize the detection of transient, less significant side effects.

## Supporting information

Riessland et al. supplementary material Riessland et al. supplementary material

## Data Availability

Due to the sensitive nature of the original data and the retrospective nature of this study, raw data should remain confidential and should not be shared.

## Supplementary material

The supplementary material for this article can be found at http://doi.org/10.1017/S1092852925100606.
