# Supplementary material for: The effects of electroconvulsive therapy on cognition: an exploratory retrospective study
Source: CNS Spectr. 2025 Oct 10;30(1):e87. doi: 10.1017/S1092852925100606 (PMC13064711; doi:10.1017/S1092852925100606)
Supplement: Riessland et al. supplementary material [file S1092852925100606sup001.docx]

**Supplementary Material**

*
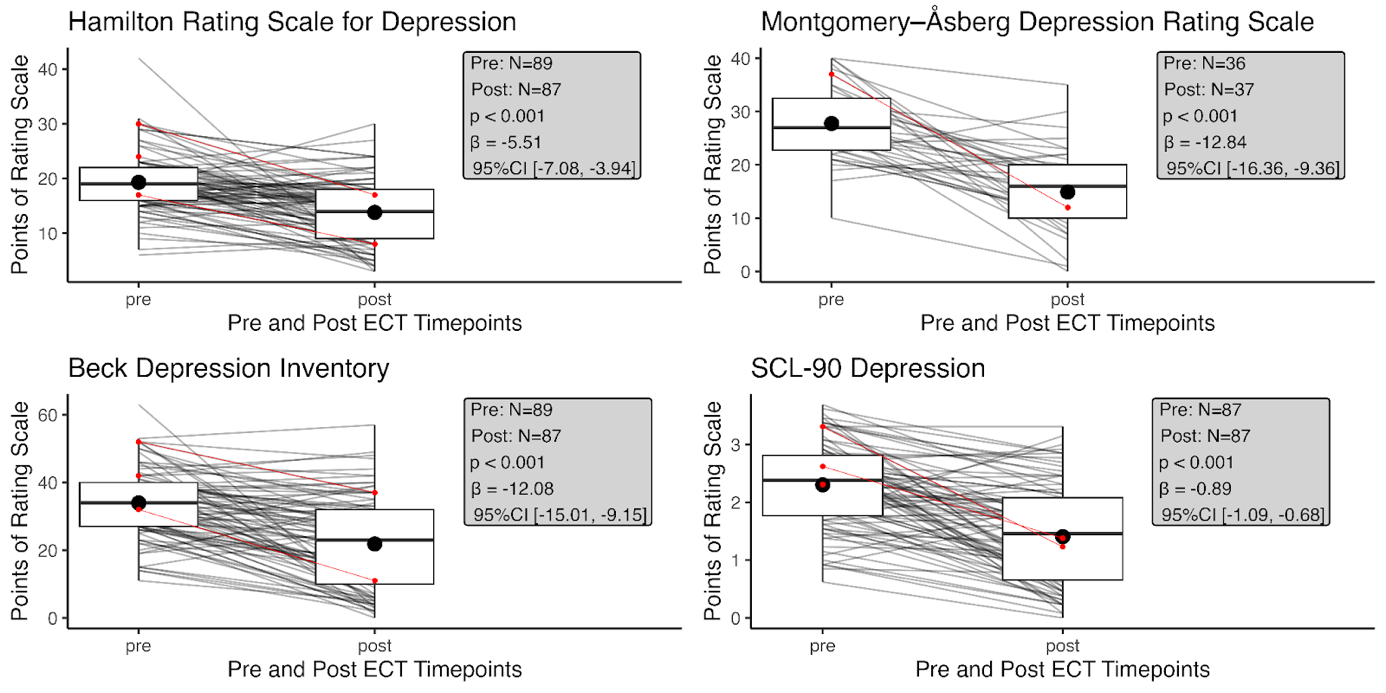
*

*Supplementary Figure 1. Title: Results from Depression Scores pre/post-ECT.*

*The central line within each box indicates the median value. The box limits illustrate the interquartile range (IQR, from the 25th to the 75th percentile). The whiskers extend to the minimum and maximum values within 1.5 times the IQR from the quartiles. Data points beyond whiskers are shown as lines. The large black dot indicates mean scores. Each grey line represents individual patients’ scores over time, with those outlined in red showing the three patients with deterioration in MMSE scores post-ECT. The number of observations pre/post-ECT, p-value, β-estimate and 95% CI are shown in the grey box for each test.*

*
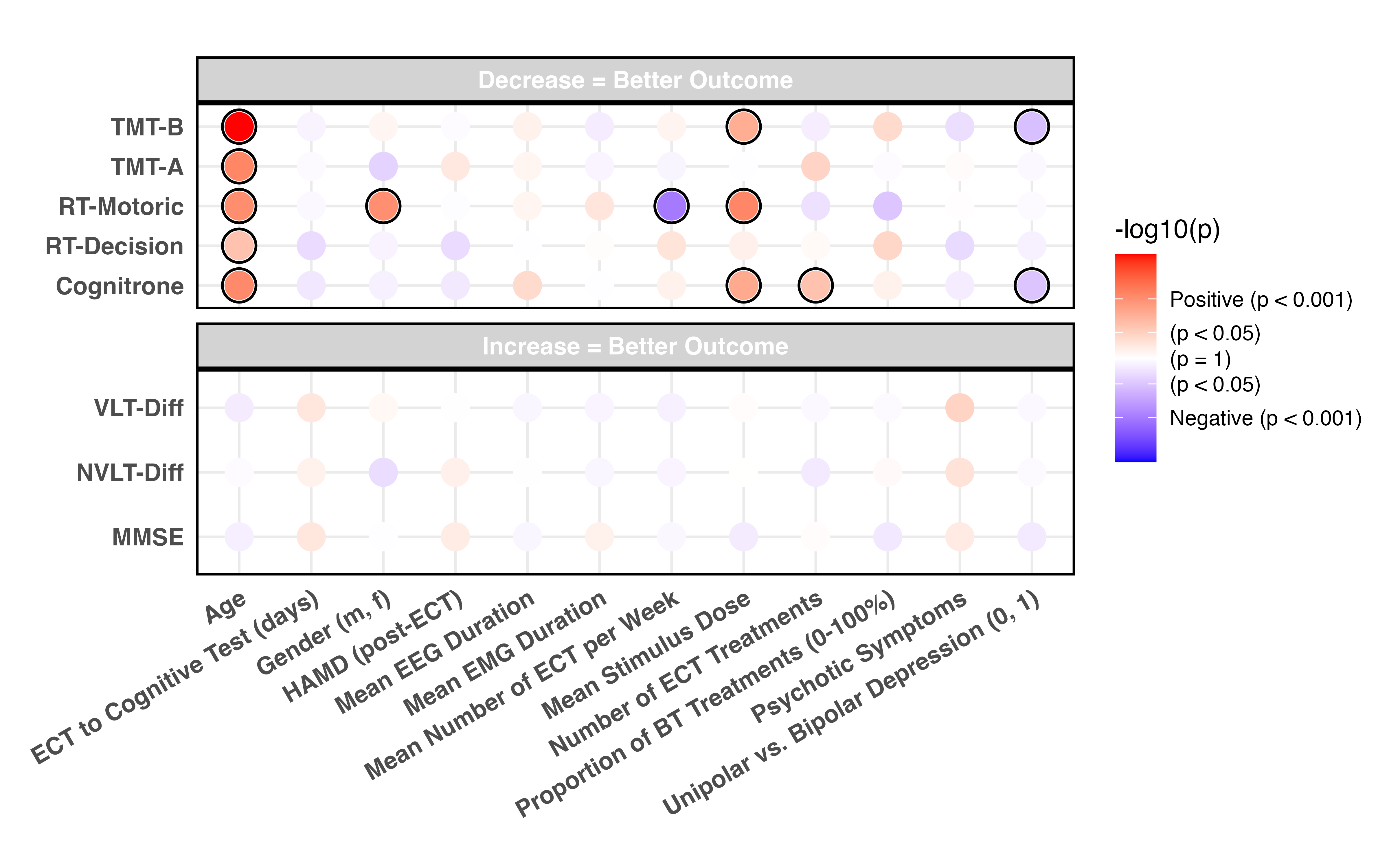
*

*Supplementary Figure 2. Title: Associations between post-ECT Cognitive and Predictive Variables.*

*All variables were assessed in a combined regression model, differing from results in Figure 2, where associations were calculated separately for each predictor variable. Each variable is corrected for pre-ECT values. Red indicates a positive association, blue a negative association, the darkness the strength of the association. In the five variables shown above, a decrease (in seconds) equals a better outcome, while in the three variables below an increase (in points) indicates a better outcome. Significant results (p-value < 0.05) are indicated by a black circle.*

|  |  |  |  |  |  | **95% CI** | |  |
| --- | --- | --- | --- | --- | --- | --- | --- | --- |
| **Variable** | **N** | **Mean (Pre)** | **Mean (Post)** | **β-Estimate** | **Effect Size** | **Lower CI** | **Upper CI** | **p-Value** |
| Δ HAMD | 87 | 19.30 | 13.79 | -5.51 | -0.86 | -7.08 | -3.94 | <0.001 |
| Δ MADRS | 36 | 27.75 | 14.89 | -12.84 | -1.28 | -16.36 | -9.36 | <0.001 |
| Δ BDI-II | 87 | 33.94 | 21.84 | -12.08 | -0.89 | -15.01 | -9.15 | <0.001 |
| Δ SCL-90 Depression | 85 | 2.30 | 1.40 | -0.89 | -0.96 | -1.09 | -0.68 | <0.001 |
| Δ STAI-State | 88 | 59.70 | 49.51 | -10.22 | -0.78 | -12.91 | -7.52 | <0.001 |
| Δ STAI-Trait | 88 | 61.61 | 52.45 | -9.14 | -0.77 | -11.78 | -6.51 | <0.001 |

*Supplementary Table 1. Difference in scores post-pre ECT of Depressive and Anxiety Variables*

|  |  |  |  |  |  | **95% CI** | |  |  |
| --- | --- | --- | --- | --- | --- | --- | --- | --- | --- |
| **Variable** | **N** | **Mean (Pre)** | **Mean (Post)** | **β-Estimate** | **Effect Size** | **Lower CI** | **Upper CI** | **p-Value** | **Outcome Direction** |
| Δ MMSE | 89 | 29.06 | 28.96 | -0.10 | -0.07 | -0.44 | 0.25 | 0.583 | ↑ = Better |
| Δ NVLT-Diff | 84 | 16.89 | 19.86 | 3.29 | 0.33 | 1.86 | 4.72 | <0.001 | ↑ = Better |
| Δ VLT-Diff | 85 | 25.92 | 22.94 | -2.86 | -0.34 | -4.87 | -0.87 | 0.006 | ↑ = Better |
| Δ Cognitrone | 85 | 3.69 | 3.73 | 0.04 | 0.02 | -0.24 | 0.32 | 0.778 | ↓ = Better |
| Δ RT-Decision | 85 | 694.78 | 711.44 | 16.84 | 0.08 | -34.33 | 67.98 | 0.518 | ↓ = Better |
| Δ RT-Motoric | 85 | 276.48 | 258.36 | -19.88 | -0.17 | -38.17 | -1.54 | 0.035 | ↓ = Better |
| Δ TMT A | 87 | 24.36 | 23.10 | -1.22 | -0.11 | -2.77 | 0.32 | 0.122 | ↓ = Better |
| Δ TMT B | 87 | 48.99 | 45.97 | -2.76 | -0.08 | -9.23 | 3.70 | 0.402 | ↓ = Better |

*Supplementary Table 2. Difference in scores post-pre ECT of Cognitive Variables*

|  |  |  | **95% CI** | |  |
| --- | --- | --- | --- | --- | --- |
| **Variable** | **Predictor** | **β-Estimate** | **Lower CI** | **Upper CI** | **p-Value** |
| MMSE | ECT to Cognitive Test (days) | 0.08 | 0.00 | 0.16 | 0.040 |
| VLT | Psychotic Symptoms | 9.08 | 0.93 | 17.22 | 0.029 |
| Cognitrone | Age | 0.02 | 0.00 | 0.04 | 0.015 |
| Cognitrone | Number of ECT Treatments | 0.17 | 0.05 | 0.29 | 0.005 |
| Cognitrone | Unipolar vs. Bipolar Depression (0-1) | -0.88 | -1.74 | -0.01 | 0.047 |
| Cognitrone | Mean Stimulus Dose | 0.01 | 0.00 | 0.02 | 0.023 |
| RT-Decision | Age | 4.55 | 0.74 | 8.36 | 0.020 |
| RT-Decision | Psychotic Symptoms | -236.46 | -446.05 | -26.87 | 0.028 |
| RT-Motoric | Age | 2.51 | 1.30 | 3.71 | <0.001 |
| RT-Motoric | Mean Number of ECT per Week | -60.23 | -107.69 | -12.77 | 0.014 |
| RT-Motoric | Gender (m, f) | 32.66 | 1.41 | 63.90 | 0.041 |
| RT-Motoric | Mean Stimulus Dose | 0.56 | 0.09 | 1.04 | 0.021 |
| TMT-A | Age | 0.18 | 0.07 | 0.29 | 0.002 |
| TMT-B | Age | 0.86 | 0.50 | 1.23 | <0.001 |
| TMT-B | Psychotic Symptoms | -23.28 | -43.89 | -2.66 | 0.027 |
| TMT-B | Mean Stimulus Dose | 0.24 | 0.09 | 0.40 | 0.002 |

*Supplementary Table 3. Significant associations between post-ECT cognitive and predictive variables, corrected for pre-ECT values for each variable.*

|  |  |  | **95% CI** | | | |  |  |  |
| --- | --- | --- | --- | --- | --- | --- | --- | --- | --- |
| **Variable** | **Predictor** | **β-Estimate** | **Lower CI** | | **Upper CI** | | **p-Value** |  |  |
| Cognitrone | Age | 0.03 | 0.01 | 0.05 | | <0.001 | | |  |
| Cognitrone | Number of ECT Treatments | 0.15 | 0.02 | 0.29 | | 0.021 | | |  |
| Cognitrone | Unipolar vs. Bipolar Depression (0, 1) | -0.83 | -1.66 | 0.00 | | 0.049 | | |  |
| Cognitrone | Mean Stimulus Dose | 0.01 | 0.00 | 0.02 | | 0.004 | | |  |
| RT-Decision | Age | 4.85 | 0.75 | 8.95 | | 0.021 | | |  |
| RT-Motoric | Age | 2.09 | 0.88 | 3.30 | | <0.001 | | |  |
| RT-Motoric | Mean Number of ECT per Week | -103.95 | -164.11 | -43.79 | | 0.001 | | |  |
| RT-Motoric | Gender (m, f) | 46.82 | 19.69 | 73.94 | | 0.001 | | |  |
| RT-Motoric | Mean Stimulus Dose | 1.05 | 0.47 | 1.64 | | <0.001 | | |  |
| TMT-A | Age | 0.23 | 0.10 | 0.35 | | <0.001 | | |  |
| TMT-B | Age | 0.91 | 0.54 | 1.27 | | <0.001 | | |  |
| TMT-B | Unipolar vs. Bipolar Depression (0, 1) | -15.99 | -30.89 | -1.08 | | 0.036 | | |  |
| TMT-B | Mean Stimulus Dose | 0.28 | 0.08 | | 0.47 | | 0.006 | | |

*Supplementary Table 4. Significant associations between post-ECT cognitive and predictive variables assessed in a combined regression model, corrected for pre-ECT values for each variable.*
